# Recent Development of Air Gauging in Industry 4.0 Context

**DOI:** 10.3390/s23042122

**Published:** 2023-02-13

**Authors:** Miroslaw Rucki

**Affiliations:** Faculty of Mechanical Engineering, Kazimierz Pulaski University of Technology and Humanities in Radom, ul. Stasieckiego 54, 26-600 Radom, Poland; m.rucki@uthrad.pl

**Keywords:** Industry 4.0, measurement, air gauging, calibration, uncertainty

## Abstract

The paper presents a review of the research reports published in 2012–2022, dedicated to air gauging. Since most of the results are somehow related to Industry 4.0 concept, the review put the air gauging to the context of fourth industrial revolution. It was found that despite substantial decrease of the number of published papers in recent years, the investigations are still performed to improve air gauges, both in static and in non-steady states. Researchers paid attention to the digitization of the results, models and simulations, uncertainty estimation, calibration, and linearization. Specific applications covered real-time monitoring and in-process control, as well as form and surface topography measurements. Proposed solutions for integration with computer systems seem suitable for the air gauges be included to the sensor networks built according to the Industry 4.0 concept.

## 1. Introduction

Pneumatic systems were for the first time used for dimensional measurements perhaps as early as in 1917 [1]. Reviewing history and future of the air gauging in 1958, Tanner stated [2]: “As electronics are increasingly used as the ‘brains’ of automatic machining, so air-operated gauges will be chosen as the ‘eyes,’ to assess and signal or send impulses, according to how the process is running”. Fifty years later, in 2008, Schuetz summarized advantages of air gauging [3]: “It is hard to imagine that a small hole (…) could be so important (…). Small electronic or eddy current type sensors may approach the size of an air jet, but nothing can match either its cost or ability to work in a wet and oily shop environment, right at the point of manufacture.” In essence, these statements are still valid and actual, and can explain why air gauges are widely used in the rapidly developing manufacturing conditions of Industry 4.0 concepts.

The expression “Industry 4.0″ was coined in 2011 to describe the digital transformation of manufacturing in terms of a fourth industrial revolution [4]. It gained popularity, even though some researchers indicated lack of a systematic approach demonstrating distinctive characteristics of a revolution in Industry 4.0 [5]. Instead, Industry 4.0 is usually described as a fusion of various concepts and technologies integrating physical, digital, and biological functions leading to fundamental transformation of the industrial processes and the associated relationships between suppliers, manufacturers, and final customers [6]. Among the main technology trends of Industry 4.0, the authors discussed nine of them, as follows:

- *Big Data and Analytics*, which covers processing of all kinds of data types and volumes through data collection, ordering, record and storage, retrieval, real-time analysis and dissemination that may extend beyond just automation and improvement of the existing processes [7];
- *Simulation* understood as the procedure of working out a model of a system, either real or hypothetical one, to describe and analyze the performance of the system, including the process of designing a model, usually a simplified set of assumptions expressed by a mathematical or logical relationship, and making the model to operate over time imitating the process consisting of interrelated elements [8];
- *Industrial Internet of Things* (IIoT), making possible to integrate Operational Technologies (OT) and Information Technology (IT) domains, through automation of industrial processes with relevance on machine-to-machine communication especially with a high volume of data involved [9];
- *Autonomous Robots* that are able to make decisions with high autonomy and to perform real-time tasks without intervention of humans [10];
- *Cybersecurity*, i.e., protection of manufacturing systems from cyber-attacks that may entail some negative impacts, such as (1) sabotage of the infrastructure or machines and components, (2) denial of networks and computers proper service, (3) crimes like theft of intellectual property, (4) violation of safety and environmental pollution, (5) dangerous and life-threatening situations for workers [11];
- *Horizontal and Vertical System Integration*, including physical and business structures, as well as integration of physical and digital worlds through cyberphysical systems, where vertical integration covers the alignment of human, equipment, organization, products, etc., and the horizontal integration interconnecting procurement, planning, management, and customer services with counterparties of a company [12];
- *The Cloud* providing virtually unlimited on-demand utilities of computing, storage, and communication resources [13];
- *Additive Manufacturing* covering a wide range of technologies that can build objects and components adding layer-by-layer portions of a raw material [14];
- *Augmented Reality* as a sort of “immersive technology”, aimed to strengthen the interaction between humans and the industrial environment, increasing the connection with surrounding objects through the intensified perception of objects without replacing the reality with virtual objects [15].

Especially four of them, namely, Additive Manufacturing, Artificial Intelligence, Internet-of-Things, and Big Data, could be identified as key technologies that promote circular product lifetimes through the improved product design, maintenance, redistribution, and recovery [16]. Thus, implementation of Industry 4.0 concepts makes an important contribution to industrial innovation and sustainability. Calabrese et al. [17] emphasized that the adoption of Industry 4.0 technologies can be seen worldwide, with robots and cybersecurity among the technologies most often reported in scientific papers. Kebisek et al. [18] indicated the necessity to incorporate technologies like Big Data and analytics, when a company wants to adapt for Industry 4.0 concepts, so that enough information can be collected to observe and analyze the ongoing manufacturing processes.

## 2. Feasibility of Air Gauging to the Concept of Industry 4.0

In the industrial processes, monitoring and control are the tools most widely applied in the frames of Industry 4.0 strategy [19]. Historically, in-process control can be considered one of the precursors of a real-time process monitoring and decision-making, and the air gauges have been widely applied in this sort of control systems [20,21]. It can be even stated that “some of the development of in-process gauging systems for turning machines has been developed along the principle of air gauging” [22]. Here, the analogue output from air gauges was used to start a servomechanism controlling the tool position, and thus the desired diameter of the component was achieved.

In his handbook, Raghavendra, among the main advantages of air gauges, pointed out the absence of metal-to-metal contact, high amplification, low cost, and ability to inspect several features at once [23]. He distinguished between the functional and metrological features, naming the advantages as follows. Among metrological features he listed non-contact inspection, minimum gauging force, possibility of variable inspection (size variability), and possibility of attribute inspection (GO/NO GO). Among functional merits he mentioned no wear of the gauge parts and workpiece, self-cleansing of the gauge tip and measured surface, rapid response, no hysteresis, remote positioning of the gauge heads, and compact size of the gauge head. In the chapter *Industrial Metrology*, Smith [24] placed the resolution of the best air gauges at the level of 10^–7^ m, i.e., 0.1 μm. It is widely recognized that air gauges are suitable measurement devices for a final check of the machines components like shafts [25], inspection of the machined pieces of the nuclear graphite [26]. More specifically, air gauges can be used also for cutting force measurement [27], for 2D roughness measurement [28] and surface topography characterization [29] including moving surfaces [30], for wheel-tooling gap measurement in the extrusion machinery [31], for continuous tool wear monitoring [32], or to control the gap between the lens and the surface in the auto-focusing mechanism, where it was able to reach an accuracy of 100 nm [33].

During the century of development of the air gauging, many reviews and summarizing papers were published on that topic [34,35,36]. In the recent decades, however, scientific interest in the air gauging substantially declined with simultaneous increase of number of reports on other, newly developed measurement methods. In the present study, it was found important to analyze the most recent research papers, limiting the time span to the years 2012–2022. In some extend, it corresponds with the observation that around 2000, Intelligent Manufacturing was primarily associated with computer-aided design (CAD), flexible manufacturing systems, expert systems, fuzzy logic, and neural networks, while the development of modern Smart Manufacturing took place around 2010 within the concept of Industry 4.0 and automation [37]. According to Baroroh et al. [22], it is imperative nowadays to implement smart manufacturing, since otherwise it is impossible to meet the rapidly growing demand for mass customized products. Due to the recent advances in both information and operation technologies (IT and OT), the era of Industry 4.0 requires that traditional manufacturing technologies empowered by artificial intelligence and by the Industrial Internet of Things (IIoT) have become “smart” at handling the incredibly complex tasks [38]. The present study is to answer the question: what does it mean for air gauging? Is it going to be abandoned, or new chance of development opens?

Industry 4.0 is considered to be the main strategy applied in order to strengthen the competitiveness of the manufacturing sector, triggered by the requirement of shortened development and innovation cycles, and driven by advancements in information, communication and automation technologies, primarily the internet of things, artificial intelligence, big data, collaborative and cooperative robots, as well as modeling and simulation [39]. Moreover, crucial roles in Industry 4.0 play such technologies as cyber-physical systems (CPS), additive manufacturing (AM), and cloud computing (CC) [40], as well as cybersecurity, computer vision and virtual reality, smart decision support systems [41]. Examples of novel application of Industrial Internet of Things technologies involve an additive manufacturing experimental setup with monitoring of both production and post-production phases with a sensor network, potentially enabling application of innovative measurement systems [42].

When industry is confronted with new technologies, a lack of legislation combined with lack of competence may become an obstacle, but also a lack of understanding of the processes design or determining which of the known solutions can be adapted to new requirements [43]. Since the technologies that belong to the context of Industry 4.0 are very recent, there are still limited data and resources able to provide extensive coverage for all aspects related to these technologies, so that it seems necessary to collect more data to provide high reliability of the models [44]. The increasing importance of continuous and online data acquisition is emphasized by many authors [45], and due to a growing need for reliable data industrial metrology is expected to be able to collect relevant information about quality characteristics of a manufactured object.

Dimensional metrology is a key issue of a manufacturing, considered one of the cornerstones of mechanical engineering [46]. In the context of Industry 4.0, distributed measuring devices and sensor networks are used rather than individual measuring instruments, so that traceable co-calibration became a challenge, and a systemic approach is required for a metrological assessment of the entire sensor networks [47]. Digitization of the manufactured products and the machines themselves and the processes in general, including the tools, fixtures, measurement instruments, etc., with high demand of non-contact metrological inspection [48]. The common principles for findable, accessible, interoperable, and reusable (FAIR) data are extended in measurement science by traceability requirements, repeatability, and reproducibility, particularly, by the concise estimation of measurement uncertainties [47,49].

Air gauges seem able to meet those demands since they still offer sufficient magnification and high reliability in tolerances measurement well beyond the capabilities of the mechanical gauges [50]. Air gauges work as comparators, evaluating difference between the actual dimension and the standard rather than performing absolute measurement, and can be applied in the Additive Manufacturing process-specific metrology and inspection methods [51]. Conventional probes and gauges are often too large to be used for the inspection of microparts. Measuring systems must use air, light, or other noncontact scanning methods to evaluate a part’s features or dimensions. 3D metrology for micro/nanodevices and features is becoming an essential element of micromanufacturing [52]. In the measurement of a hole size between the tight fit plug and the loose one in the turbine blade, air gauging could provide an efficient measure of holes, but this method works well in a very short range of hole diameters [53]. Moreover, it is unable to detect important details of the defects of the holes. Even though air injection systems can be used in aerosol-assisted processes for submicron particle synthesis [54], they do not work here as a dimensional measurement device. Nevertheless, there are several reports on air gauging applications in the AGILE systems (Air Gauge Improved Levelling) to correct location of a die in the focal plane of the projection lens, to reduce process dependency of a lithography scanner control [55]. AGILE application is a commercial system introduced by ASML, so that details are not available on how it operates, but it provides measurement correction for the level sensor [56]. Dong et al. [57] adapted AGILE method to improve the optical level sensor by compensating the optical effects. In their approach, the scanner was allowed to generate corrections of the “optical-to-air-gage” focus for every lot or every exposed wafer. Despite some limitations, the authors found that the application of AGILE in 40 nm Via-PH process can be useful for the lithographic technology node, where calibration of the optical level sensor was performed with an air gauge sensor with AGILE system.

## 3. Research Directions in Recent Papers

Directions of the research on air gauges are highly determined by the nature of the medium used. In the pneumatic comparators, the air is a means of measurement, so that changes in the measured component’s feature is reflected by the changes in a calibrated flow. It is indicated in [23] that the air gauges can be classified as back-pressure gauges that were developed first, and free flow gauges that are in greater use. However, the research papers published in the last decade, from 2012 to 2022, concern exclusively with the back-pressure air gauges, as it can be seen from the below review.

**Figure 1:**
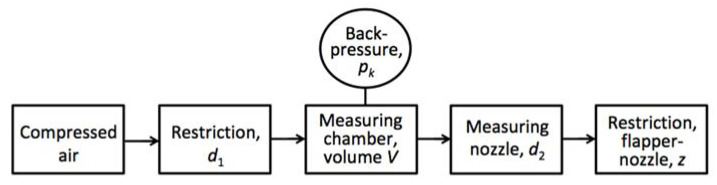
Block diagram of a typical back-pressure air gauge.

The main factor determining the performance of a back-pressure air gauge is the air flow through its elements, such as inlet and measuring nozzle, measuring chamber, and flapper-nozzle area. Typical cascade arrangement of these components is shown in Figure 1.

The compressed air flows from the compressor to the flapper-nozzle area, where it is released to the atmosphere. In the open jest systems, the measured surface plays the role of flapper directly. The back-pressure in the measuring chamber depends directly on the displacement *z* between the nozzle and the restricting surface, determined by the dimension of the measured element. In certain area, graph of the back-pressure *p_k_(z)* is proportional, which is used as a measuring range. Usually, the setting points of the static characteristics are calibrated using the setting masters of known dimensions.

### 3.1. Researches on Static Characteristics of the Air Gauges

When projecting and using air gauges, it is necessary to take into consideration main properties of the compressed air, thermodynamic processes, and some laws of the fluid mechanics in respect of the geometry of the flow-through components [58]. Jermak [59] undertook a discussion on the complex phenomena pointing out the supersonic areas and appearance of a vacuum ring in the flapper-nozzle area, which caused discontinuity and hysteresis in the metrological characteristics. The author analyzed also the expansion of compressed air stream in the measuring chamber, right after the inlet nozzle. Based on the results, he proposed some recommendation on the geometrical parameters of the nozzles, as well as on the optimal localization of the pressure transducer in the measuring chamber. Burazer et al. [60] investigated influence of the inclination of a measuring nozzle on pressure distribution on the measured surface, and the respective effect on the air gauge sensitivity and its measurement range. The authors demonstrated that the inclination had a positive effect on the air gauge performance, even though they did not find any clear trend regarding the changes in sensitivity caused by the measuring nozzle inclination. Analyzing the vacuum zone in the flapper-nozzle area, the researchers found a displacement of the vacuum zone away from the nozzle’s longitudinal axis. This means that the impurities gathering will be reduced. From the point of view of pneumatic metrology application, measuring nozzle inclination is beneficial since it leads to a decrease in the size of the vacuum area. Another positive effect that is achieved with nozzle inclination is the translation of the vacuum zone away from the measuring nozzle’s axis.

**Figure 2:**
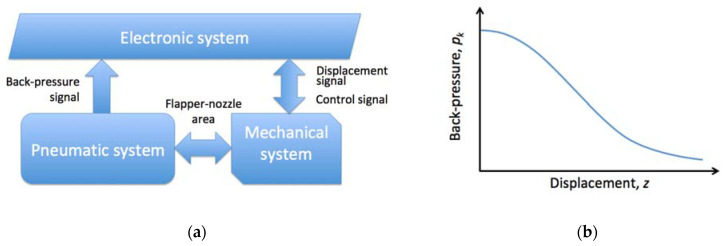
Schematic of the test rig: (**a**) Simplified block diagram of the air gauge test rig; (**b**) Registered relation between displacement *z* and back-pressure *p_k_*.

For the experimental determination of the air gauge static characteristics, Jakubowicz and Derezynski [61] proposed an automated, computerized laboratory rig. They distinguished between three systems: pneumatic, electronic, and mechanical ones. Flapper-nozzle area is the place of interaction between mechanical and pneumatic systems, and the electronic system collects signals from both of them with simultaneous performing necessary control functions. The working principle can be explained by a simplified diagram shown in Figure 2.

The authors explained that the properly prepared compressed air flows to an air gauge passing an air flow meter, and apart from the back-pressure measurement, air temperature and mass flow is monitored. The air gauge may represent any sort of constructional structures, but primarily, the test rig was designed for analysis of an open-jet straight cascade gauge, where compressed air passed from a feeding chamber through the restriction to the measuring chamber, and then through the measuring nozzle and flapper-nozzle slot to the atmosphere. Both the inlet and measuring nozzles are replaceable to test the air gauges of the desired metrological parameters, such as multiplication and measuring range. The authors applied dedicated software that has been worked out in their team, with intuitive user interface that allows real-time control of the measurement process tracking the values on-line. As such, the developed test rig with the experimentally determined air gauges characteristics can be successfully integrated to the IoT and big data analysis, crucial for Industry 4.0 concept.

There are many models for air gauge performance estimation, proposed throughout the 20th century around the World. One of the possibilities is calculation of the air flow using air-electric equivalence method, i.e., considering equivalent the air flow and electric current. The authors [62] assumed equivalency of air flow’s potential energy represented by air flow pressure and the potential energy of the electric current represented by voltage. Thus, they calculated the static characteristics using the equation:

(1) $$p_{k}(z)=\frac{P}{{\left(\frac{4d_{2}}{d_{1}^{2}}\right)}^{2}z^{2}+1},$$

where *P* is the air source pressure, parameters *d*_1_, *d*_2_ are control orifice and measuring nozzle diameters, respectively, and *z* is a gap in the flapper-nozzle area. The accuracy of this steady-state approximation was proved sufficient for the evaluation of grinding wheel wear in wet profile grinding for the groove of the ball bearing’s inner ring.

Due to the complexity of the thermodynamic phenomena in the air gauge cascade, many researchers combined theoretical formulas with experimental results [63]. In a steady-state empirical-mechanistic modeling, the mathematical calculations based on available theoretical background were combined with the empirical polynomial approximating functions of the first and second orders [64]. Jermak et al. [65] compared several models, usually used for the calculation of air gauge characteristics, such as the one proposed by Balakshin [66], Gluchow [67,68] or the model based on Saint Venant-Wenzel equations. The latter approach distinguished between different flow conditions in the pneumatic cascade, where sonic air flow velocity can be reached independently in the inlet restriction (I) and the outlet flapper-nozzle area (II). The following combinations of the pressure rates are possible:

- In both areas I and II the pressure ratio is below *β_kr_*;
- In the inlet restriction (I) the pressure ratio is below *β_kr_*, while in the flapper-nozzle area (II) it is higher than *β_kr_*;
- In the area I the pressure ratio is higher than *β_kr_*, while in the area II it is lower;
- In both areas I and II the pressure ratio is higher than *β_kr_*.

Depending on the conditions above, relations of air flow coefficients *α* and pressure ratios would look different. For instance, when the pressure ratio is below *β_kr_* in both areas I and II, i.e., in the inlet restriction and the outlet flapper-nozzle area, the equation could be written as follows [65]:

(2) $$y=\sqrt{{\left(\frac{bx^{2}-1}{2}\right)}^{2}+{\left(bx\right)}^{2}}-{\left(\frac{bx^{2}-1}{2}\right)}^{2},$$

where *b = p_a_*/*p_k_* is the normalized steady-state back-pressure, i.e., ratio of the atmospheric pressure *p_a_* to the back-pressure *p_k_*; *x* denotes the relation between flow coefficients and respective flow-through areas of inlet nozzle and flapper-nozzle slot, as follows:

(3) $$x=\frac{\alpha_{2}}{\alpha_{1}}\frac{A_{2}}{A_{1}},$$

where air flow coefficients *α*_1_ and *α*_2_ correspond with inlet restriction and flapper-nozzle restriction of respective flow-through areas *A*_1_ and *A*_2_. The latter, in turn, depends on respective nozzle diameters *d*_1_ and *d*_2_, as well as on the actual displacement *z*, as shown schematically in Figure 1.

However, in other cases the Equation (2) becomes more simple, and when the pressure ratio is higher than *β_kr_* in both areas I and II, this formula looks as follows:


(4) $$y=\frac{1}{x}.$$

The authors [65] from their investigations proposed also a model based on the measurement of the actual mass flow ${\dot{m}}_{actual\;max}$, which appeared to be smaller than the one calculated theoretically due to the stream contraction and respective losses of pressure. They used term ‘second critical parameters’ to describe the difference between the pressure ratio for actual maximal mass flow and the theoretical one, as follows:


(5) $$\alpha_{kr2}=\frac{{\dot{m}}_{actual\;max}}{{\dot{m}}_{theor\;max}}.$$

Thus, their model denoted SCP (for Second Critical Parameters) underwent comparison with other ones. The performed simulations provided calculated characteristics very close to the empirical ones, with differences below 5%, while the relative error of other models exceeded 10%, especially for larger displacements. It should be noted that the demonstrated creation of advanced models and simulation of the behavior of the systems belongs to the Industry 4.0 enabling technologies, strongly supporting the manufacturing process design [69].

### 3.2. Investigations on Dynamic Characteristics

**Figure 3:**
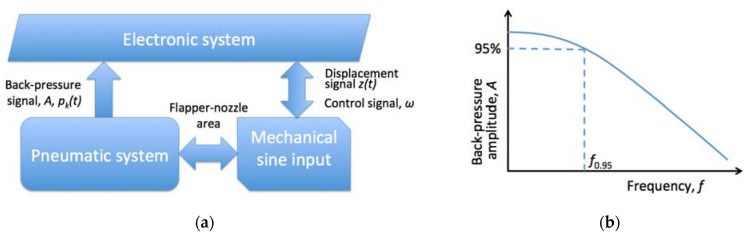
Schematic of the dynamic calibration: (**a**) Simplified block diagram of the air gauge dynamic calibration setup; (**b**) Registered amplitude-frequency characteristics.

Dynamic properties of the air gauges were considered their weak side for the long time. However, it was demonstrated that under certain conditions, time constant of the air gaging system was improved to reach several milliseconds [70]. It became possible through the constructional changes, with application of the piezoresistive pressure transducers directly in the reduced volumes of the measuring chamber. The block diagram of dynamic calibration system is shown in Figure 3a. It is essentially similar to the system shown in Figure 2, but the input value of slot width *z* was sinusoidally changed with preset frequency, which was obtained through eccentric shaft and its rotating frequency *ω*. The output back-pressure signal was analyzed in amplitude terms, so that amplitude-frequency characteristics were obtained, as shown in Figure 3b. Graphically, it can be seen that under the input frequency *f*_0.95_, back-pressure amplitude *A* reached only 95% of its static value *p_k_*.

Jermak et al. [71] performed the analysis of various mathematical models for dynamic characteristics of the air gauges, where the back-pressure is time-dependent *p_k_(t)* due to the unsteady state of input value *z(t)*. They found that the most accurate approximation could be made using second critical parameters of the air flow, dependent primarily on the geometry of the nozzles applied in the actual configuration of the air gauges.

Jakubowicz et al. [72] analyzed effect of the inlet nozzle diameter on the dynamic performance of an air gauge. As it had been expected, dynamics of the air gauges worsened when smaller inlet nozzles were applied, increasing time constant up to 100% in some cases, e.g., when diameter of the inlet nozzle was reduced form 1.210 mm down to 0.720 mm. The authors concluded, that the time constant *T* values between 0.04 and 0.13 s could be obtained, with the dynamic error *f*_0.95_ < 5% at the sine input frequencies range from 3 to 8 Hz. However, the steepest improvement technique reported earlier [73] proposed for the never-ending improvement of the dynamics of air gauges, demonstrated progressive improvement and reduction of the setting time from 118 to 10 ms.

### 3.3. Uncertainty Estimations for Air Gauging Systems

The increase of information should be connected with the reduction of uncertainty level, that is why Industry 4.0 concept requires a particular focus to be made on the uncertainty budget [74]. The uncertainty of an air gauging, and, specifically, of a respective test rig has been investigated by Jermak and his research team [75]. In the analysis, it was distinguished between the mechanical, electronic, and air supply systems. In the case of environmental effect, it was determined that the controlled laboratory conditions were able to minimize it, since the laboratory was isolated from vibrations, while ambient pressure, humidity, and temperature were measured constantly. As for the operator’s influence on the measurement results, it was reduced to the fixation of the examined air gauge, zero setting and initial data input, while the entire measurement process was fully automated. The authors estimated separately uncertainties for the mechanical test rig system, reference inductive gauge, back-pressure indications, and data processing. They obtained the results with the largest expanded uncertainty (level of confidence 95%) for a single measurement *U*_0.95_ = 0.260 kPa, which corresponded with the dimensional measurement of 0.52 μm. Finally, equipment variation *EV* was calculated, covering uncertainties of both test rig and the tested air gauge. It included deviations for different configurations of the air gauges and for different points in its static characteristics.

Similarly, uncertainty estimation for the dynamical characteristics were assessed and reported in [76]. The dynamic calibration apparatus used in the research generated mechanical sine input with an eccentric shaft, so that different input frequencies could be set. The experiments were performed for different volumes of the air gauge measuring chambers, from *V*_min_ = 0.5 cm^3^ up to *V*_max_ = 4.0 cm^3^. The authors distinguished three main sources of uncertainty: one related to the pneumatic system (air supply, geometry of the air gauge components, pressure transducer, etc.), other related to the mechanical system generating the periodically changing slot, and the third one related to the data processing (averaging, smoothing algorithms, interpolation, linearization, etc.). From the obtained amplitude-frequency graph, the maximal frequency *f*_0.95_ was determined, which indicated the working frequency when the dynamic error of the amplitude did not exceed 5%. Since the obtained results exhibited distribution close to the Gaussian one, Type A standard uncertainty *u(x)* was assumed to be equal to the standard deviation *σ*, according to the JCGM recommendations [77]. The authors calculated expanded uncertainties for coverage factor *k* = 2, which corresponded with 95% confidence level, for time constant ±0.001 s, and ±0.0018 Hz for frequency *f*_0.95_.

### 3.4. Recent Specific Applications of the Air Gauges

Tanaka and Koshy [78] demonstrated feasibility of a pneumatic sensor application for in-process monitoring of the performance of grinding wheels. The nominal distance between the measuring nozzle surface and the wheel periphery was 75 μm. The authors used smaller nozzle diameters due to a higher dynamic sensitivity, but their application appeared to be limited because of the porous structure of the monitored wheels. The frequency characteristics of the back-pressure signals indicated distinct peaks at multiples of the grinding wheel rotational frequency, at speeds of up to 30 m/s. A comparison with a numerical model simulations confirmed that the registered signal indeed corresponded with a wheel topographic features like grains and pores. Further assessment was made in terms of monitoring of progressive wheel wear and grinding burn, for vitreous and metal-bonded grinding wheels. It was also proved that the air gauging process was not affected when a flood grinding fluid was applied.

Stalin John et al. [79] described the experimental setup for a burnishing process, where penetration allowance was measured by the air gauge, recorded, and then processed by the FANUC program. For every single trial, the penetration allowance was fine adjusted and again measured with air gauges, while the bore sizes after burnishing were measured by another digital air gauges. The measurement results were processed and used for the real-time control of the process and the response optimization.

Yen et al. [80] built and on-line quality inspection system based on air gauging, designed for automotive component manufacturing process. They extracted signals from manufacturing process using a real-time monitoring module with subsequent analysis of the feature values. The authors distinguished three development steps: (1) Signal processing and feature extraction; (2) Quality inspection rule and threshold establishment; (3) Validation. The experiments were conducted to collect the data on vibrations and control signal, and to compare it with the corresponding quality inspection data from the air gauge in order to find the correlation between vibration and cutting accuracy. Using big data from a history data set, authors defined rules and thresholds are for online precision diagnosis, and implemented system by the integration of each subsystem to the real production line for validation. The experimental results covered 7286 workpieces and proved capability of the proposed system.

One of the important applications of the air gauges is the roundness assessment, due to ability to perform non-contact measurement and to work under difficult conditions. However, their application in the measurement of cylindrical parts waviness parameter is still limited [81]. There are some reports, though, demonstrating that in some conditions waviness of the profile *Wt* can be measured relatively accurately, with relative error of 5% [82]. Jakubowicz [83] proposed and investigated a roundness measuring air gauge with slot-shaped measuring nozzle. To assess ability of the as-modified air gauge, the author made a quantitative comparison with a reference method, calculating a relative error *δRONt* represented by a ratio of the difference between averages of pneumatic and stylus measurement results:

(6) $$\delta RONt=\frac{{\bar{RONt}}_{\left(PNEU\right)}-{\bar{RONt}}_{\left(HOMMEL\right)}}{{\bar{RONt}}_{\left(HOMMEL\right)}}\cdot 100\%,$$

where ${\bar{RONt}}_{\left(PNEU\right)}$ and ${\bar{RONt}}_{\left(HOMMEL\right)}$ are roundness deviations calculated according to the standard ISO 12181-2011 for least squares fit of points collected with the tested pneumatic system and reference Hommel-Etamic roundscan 535 system, respectively. The author concluded that the smallest registered relative error *δRONt* = 0.1% demonstrated that the air gauges with slot-shaped measuring nozzle revealed almost the same accuracy of roundness assessment as the stylus method. However, he pointed out the small measuring ranges below 300 μm as a limitation of its application for larger values of measured out-of-roundness. Jermak and Rucki [84] used air gauges for the measurement of the cylinder out-of-roundness with the tolerance of 10 ÷ 15 μm. After the initial calculations and choice of the air gauges parameters, their specific characteristics underwent experimental verification, so that the final configuration exhibited multiplication |*K*| = 0.5 kPa/μm and the measuring range *z_p_* = 100 μm, ensuring non-linearity below 0.5%. They demonstrated that the device was able to measure inner diameters up to 100 mm with the satisfactory uncertainty of ±1.5 μm. The automatic device was proposed based on a three-point roundness assessment [85]. It applied a novel floating gauge head with three independent air gauges, which may be compared conceptually with the V-block method. In that case, however, two air gauges played the role of two fixed points of a V-block. Using the simulations, the authors established that the random errors had very small impact on the measurement results, and as such, they may be omitted. The authors demonstrated that the relative error of the measurement results obtained from their novel system was below 10%, compared to the reference Taylor-Hobson Talyrond 365 device.

Damir et al. [86] performed some experiments to inspect the surfaces with complex geometry of truncated groove and milled surfaces at different speeds. From the registered air gauge response, the authors deduced relation between back-pressure and displacements, demonstrating good agreement between theoretical calculations and experimental results. The proposed approach as a pattern recognition technique could be used to detect the surface dimensional features, which could be beneficial for the real time process monitoring, as well as inspection of the machined surface. The reference to the CAD model for online corrective action and tool path modification in multi-axis manufacturing

### 3.5. Integration with Computer Systems

Due to the easy conversion of back-pressure signal to digital form, air gauges can be integrated with the computers to create an advanced computer systems [87]. Jermak proposed a novel Pneustar measurement device [88] consisting of three modules: a pneumatic system, electronic module, and a computer. The inlet nozzle was made in form of a controllable needle restrictor in order to adjust multiplication to the actual measuring task. A set of the theoretical static characteristics was introduced to the computer, so that calibration could be made using only one setting ring. Respective linearized characteristics of the exchangeable measuring head are recorded through the calibration table, and can be easily recalled. On the other hand, linearity improvement in larger range was possible through application of three or more setting masters, thus, in fact, stitching together digitally several sections of the same air gauge characteristic [89]. The authors demonstrated that this procedure prolonged the measuring range by 50% retaining the linearity error on the level of 1%. These features with further data processing possibilities can be considered prerequisites necessary for Industry 4.0 applications. With them, air gauge can be integrated to IoT device system possessing data storage, processing ability, system software, operating systems, preloaded applications, and communication resources [90].

## 4. Concluding Remarks

This review of the recent research reports devoted to the air gauging provided several important observations. First of all, despite significant decrease of the number of papers, the investigations are still performed to improve air gauges and, what’s more important, to make them feasible to the Industry 4.0 concept and technologies. Notably, the investigations focused on the back-pressure air gauges. In particular, reports cover several important specific applications, such as measurement of form or topography features, monitoring of the performance of grinding wheels, real-time control of the burnishing process, etc. Secondly, both static and dynamic characteristics are investigated to reach better parameters and to obtain better mathematical models. In particular, application of simplified models with satisfactory accuracy and more exact models combining theoretical formulas with experimental results is presented and justified.

The researchers demonstrated that the open jet back-pressure type air gauges ensure high accuracy retaining all the well known advantages of pneumatic measurement, such as lack of mechanical contact and friction, self-cleaning of the measured surface, or indifference toward ambient work conditions. For the air gauging systems integrated with electronic transducers and computer systems, it was demonstrated that steady-state measurement uncertainty could be below ±0.52 μm, which makes them feasible for the wide range of industrial measurement tasks. Achievable time constants of several dozens milliseconds are good enough for the most of dynamic measurements and real-time process monitoring.

In the analyzed papers, most of the Industry 4.0 constituent technologies are somehow addressed and connected with air gauging. Keeping in the first place the accuracy, linearity, and dynamic performance of the air gauges as industrial measurement device, authors increasingly connect them with big data analytics, modeling and simulations, integration with Information Technology into IIoT systems. It was demonstrated that the air gauges can be integrated with the industrial information system, and the measurement real-time signals can be adapted by the decision-making autonomous robots and stored for further analysis. Moreover, air gauges can be applied in the Additive Manufacturing process-specific metrology and inspection systems.

Very important for Industry 4.0 implementations is digitization of the measurement results with further data processing in the frames of sensor network. The analyzed publications indicated this direction of research and proposed relevant devices. In particular, the proposed solutions enabled digital linearization, simplified calibration methods, and automatic recall of the recorded characteristics. Thus, it can be concluded that the good old air gauging method has successfully entered the fourth industrial revolution era.

It can be expected that in the nearest future, reports on attempts to integrate the air gauges to the existing or developed industrial will be published in the context of maturity and readiness of Industry 4.0 implementation. It also seems inevitable that some investigations will be directed to artificial intelligence applications in processes of filtration and interpretation of the air gauge measurement signal. Perhaps in further stages of Industry 4.0 implementation in the enterprises, algorithm verification, machine-readable calibration, application of Metrology Cloud technology and other challenging interoperability issues will rise.

## Data Availability

Not applicable.
